# Genome-wide association study of agronomic traits related to nitrogen use efficiency in Henan wheat

**DOI:** 10.1186/s12864-023-09922-0

**Published:** 2024-01-02

**Authors:** Zaicheng Zhang, Chaojun Peng, Weigang Xu, Yan Li, Xueli Qi, Mingzhong Zhao

**Affiliations:** 1 State Key Laboratory of Crop Genetics and Germplasm Enhancement, Nanjing Agricultural University, Nanjing, 210095 People’s Republic of China; 2 Institute of Crops Molecular Breeding, National Engineering Laboratory of Wheat, Key Laboratory of Wheat Biology and Genetic Breeding in Central Huanghuai Area, Ministry of Agriculture, Henan Key Laboratory of Wheat Germplasm Resources Innovation and Improvement, Henan Academy of Agricultural Sciences, Zhengzhou, 450002 People’s Republic of China; 3 The Shennong Laboratory, Zhengzhou, 450002 People’s Republic of China

**Keywords:** Wheat, Nitrogen use efficiency, GWAS, RNA-seq, Candidate genes, 3VmrMLM

## Abstract

**Background:**

Nitrogen use efficiency (NUE) is closely related to crop yield and nitrogen fertilizer application rate. Although NUE is susceptible to environments, quantitative trait nucleotides (QTNs) for NUE in wheat germplasm populations have been rarely reported in genome-wide associated study.

**Results:**

In this study, 244 wheat accessions were phenotyped by three NUE-related traits in three environments and genotyped by 203,224 SNPs. All the phenotypes for each trait were used to associate with all the genotypes of these SNP markers for identifying QTNs and QTN-by-environment interactions via 3VmrMLM. Among 279 QTNs and one QTN-by-environment interaction for low nitrogen tolerance, 33 were stably identified, especially, one large QTN (*r*^2^ > 10%), *qPHR3A.2*, was newly identified for plant height ratio in one environment and multi-environment joint analysis. Among 52 genes around *qPHR3A.2*, four genes (*TraesCS3A01G101900*, *TraesCS3A01G102200*, *TraesCS3A01G104100*, and *TraesCS3A01G105400*) were found to be differentially expressed in low-nitrogen-tolerant wheat genotypes, while *TaCLH2* (*TraesCS3A01G101900*) was putatively involved in porphyrin metabolism in KEGG enrichment analyses.

**Conclusions:**

This study identified valuable candidate gene for low-N-tolerant wheat breeding and provides new insights into the genetic basis of low N tolerance in wheat.

**Supplementary Information:**

The online version contains supplementary material available at 10.1186/s12864-023-09922-0.

## Background

Bread wheat (*Triticum aestivum* L.) serves as a primary source of carbohydrates for over two billion individuals globally [1–4]. Although nitrogen (N) is the most important mineral element in the determination of wheat yield [5], N fertilizer is main N source in wheat growth and development. In the past 50 years, the growth rate of nitrogen application in wheat far exceeds that of wheat yield, especially with a nitrogen fertilizer utilization rate of less than 50% [6]. Excessive nitrogen application not only affects ecological environment but also reduces production efficiency [7, 8]. Therefore, a major challenge in crop breeding today is how to improve production efficiency by increasing nitrogen utilization efficiency (NUE).

To address the above issue, the most effective way is to breed “truly green” wheat varieties with high-NUE. However, plant NUE involves a series of physiological processes, including N sensing, uptake, translocation, assimilation, and remobilization [9], while it is a complex trait composed of numerous interacting genetic and environmental factors. Thus, it is critical to identify as many genes related to NUE as possible in the breeding of NUE wheat varieties.

Recently, genome-wide association studies and linkage analysis have successfully identified a series of quantitative trait nucleotides (QTNs) and quantitative trait loci (QTLs) for NUE-related traits in wheat [10–12]. However, most previous studies involved small number of SNP markers, small population sizes, and experimental environments deviating from production conditions, resulting in their low values in wheat breeding [13–18]. Therefore, it is necessary and important to mine NUE genes in wheat germplasms.

Grain yield per unit area (GYA) is a comprehensive reflection of crop productivity and the basis for calculating NUE [19]. NUE, defined as grain yield per unit of available N from the soil, can be is composed of nitrogen uptake efficiency (NupE) and nitrogen utilization efficiency (NutE) [20]. Effective panicle number per unit area (EPN) is not only an important component of yield, but also one of the most related traits to wheat NUE [21]. Plant height (PH) significantly affects crop GYA, and has a significantly genetic correlation with NUE [22, 23]. At present, NUE related traits during the maturity stage have been widely used in identifying rice NUE related loci [9, 24, 25]. Although Henan Province is the largest wheat producing province in China, the genes for wheat NUE related traits have not been fully mined.

To address the above issues, 244 common wheat accessions in Henan Province were genotyped by 660 K wheat microarray and phenotyped by three NUE-related traits (GYA, PH, and EPN) at maturity under high nitrogen (HN) and low nitrogen (LN) conditions in the field in three successive years. 3VmrMLM [26] was used to identify QTNs and QTN-by-environment interactions for the three traits. Around these significant loci, candidate genes were predicted based on RNA-seq analysis. This study provides a basis for the further Investigation to detect causal genes of these traits in the future.

## Results

### Phenotypic data analysis

**Fig. 1:**
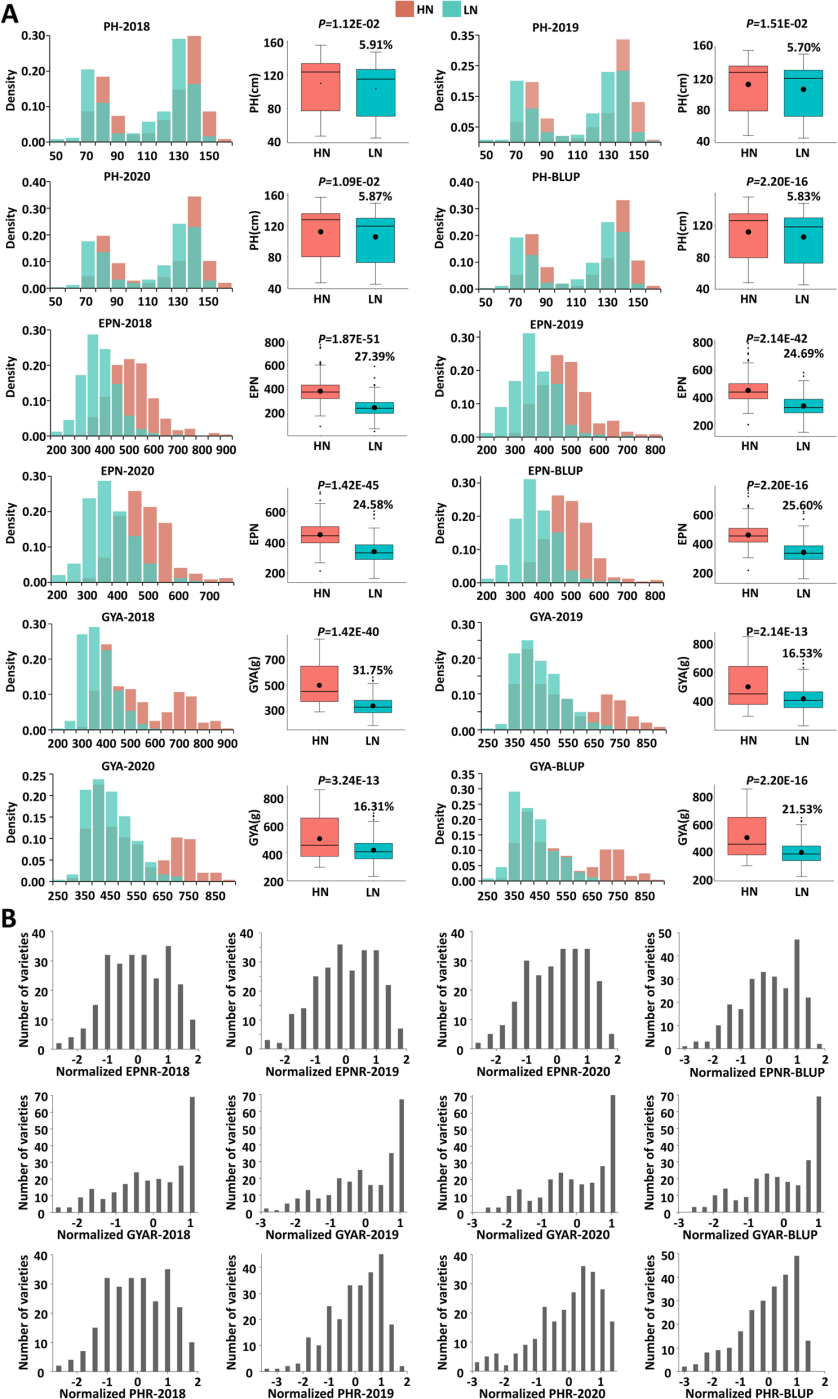
Phenotypic diversity of 244 wheat accessions. **A** Frequency distributions and differences for PH, EPN, and GYA of the wheat accessions measured under LN and HN in 2018, 2019, 2020, and the BLUP across all three environments. PH: plant height, EPN: effective panicle number per unit area, GYA: grain yield per unit area. BLUP: Best linear unbiased predictions. Box edges represent the 0.25 and 0.75 quantiles with the median values shown by bold lines. Whiskers extend by no more than 1.5 times the interquartile range, and the remaining data are indicated by dots. *P* values were calculated with Student’s *t* test. **B** Frequency distribution histogram of zero mean normalized phenotypic values of PHR, EPNR, and GYAR in 2018, 2019, 2020, and BLUP. PHR: plant height ratio of the low to high nitrogen treatments (LN/HN); EPNR: effective panicle number ratio of LN/HN; GYAR: grain yield per unit area ratio of LN/HN; 2018, 2019, and 2020: the 2017–2018, 2018–2019, and 2019–2020 field experiments, respectively

Three NUE-related traits, PH, EPN, and GYA, for all the 244 accessions were measured under the HN and LN conditions in the field in 2017–2018, 2018–2019, and 2019–2020 (Table 1 and S1). The results showed that, under the LN treatment, PH, EPN, and GYA were decreased only by 6%, 26%, and 22%, respectively, indicating the effectiveness of the LN treatment (Fig. 1A). To reflect NUE, we focused on the ratio of the trait value under LN to the trait value under HN. PH ratio (PHR), EPN ratio (EPNR), and GYA ratio (GYAR) were then calculated for each accession in each year. The frequency distributions for all three NUE-related trait ratios showed a continuous distribution across all the three environments and BLUP values, coefficients of variation for the BLUP values in three environments ranged from 17.00% (EPN) to 29.53% (GYA) under the HN treatment and from 19.47% (GYA) to 26.97% (PH) under the LN treatment (Table S2), indicating the suitability of these traits for conducting genome-wide association studies (Fig. 1).

**Table 1:** The soil basal fertility and fertilizer application rate for each treatment in 2017–2018, 2018–2019, and 2019–2020

Year	Treatment	Determination parameter (kg⋅ha^−1^)			Base fertilizer (kg⋅ha^−1^)			Topdressing N (kg⋅ha^−1^)
		**Alkali hydrolysable N**	**Available phosphorus**	**Available potassium**	**N**	**P**_**2**_**O**_**5**_	**K**_**2**_**O**	
2017-2018^a^	HN	249.10	116.10	440.90	171.00	129.30	45.00	69.00
	LN	216.90	117.50	447.70	0.00	129.30	45.00	0.00
2018-2019^a^	HN	228.60	130.20	561.20	140.30	120.00	75.00	69.80
	LN	218.90	128.40	564.20	0.00	120.00	75.00	0.00
2019–2020	HN	238.90	123.20	501.10	156.70	125.20	69.30	69.40
	LN	217.90	123.00	506.00	0.00	125.20	69.30	0.00

^a^Information of the soil basal fertility and fertilizer application rate for each treatment in 2017–2018 and 2018–2019 from Peng et al. (2022) [13]

### Genotype data, LD decay, and population structure analysis

**Fig. 2:**
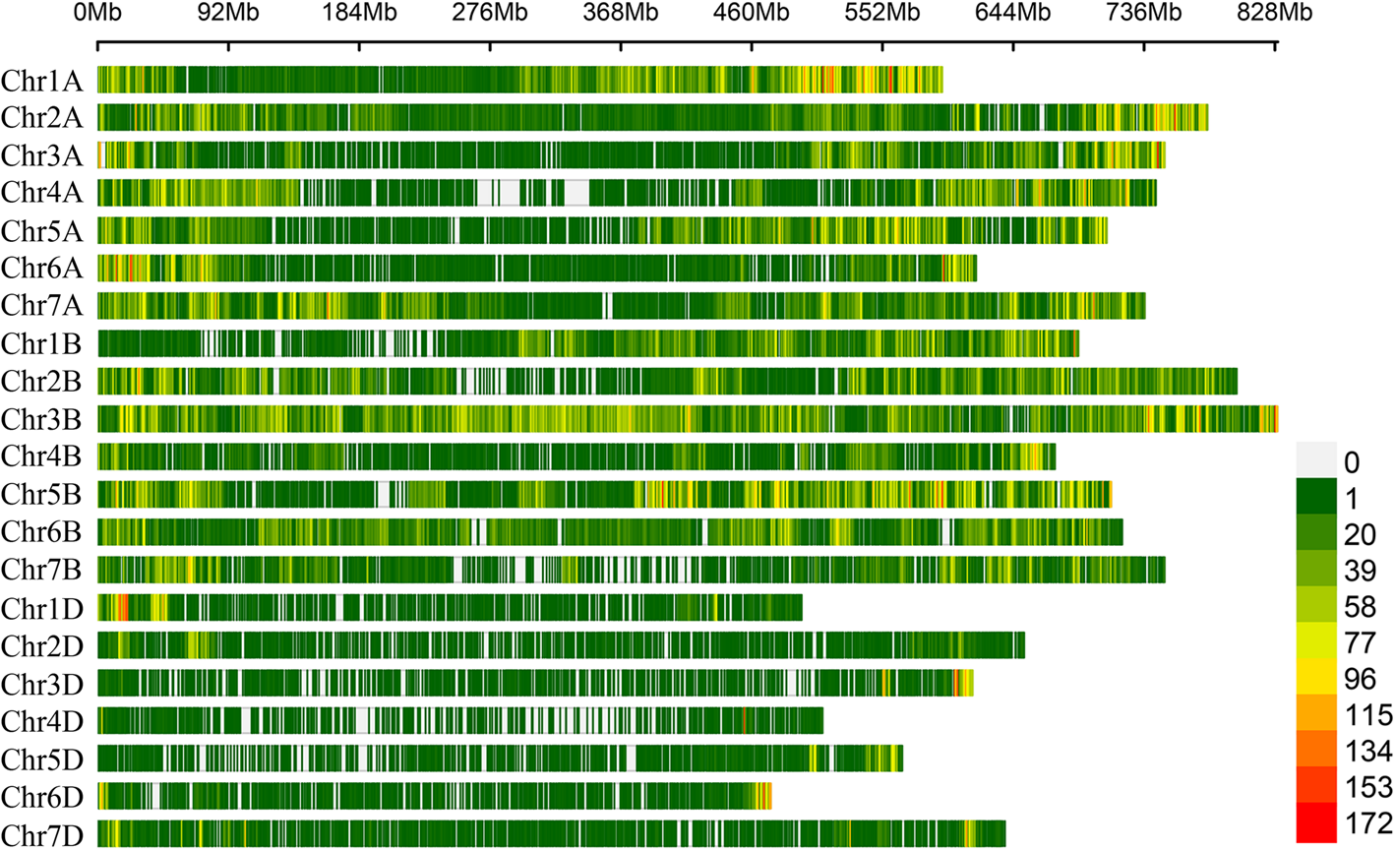
The density distribution of single nucleotide polymorphisms (SNPs) in wheat. Red and gray horizontal bars show genomic regions that are rich and poor in SNPs, respectively

After quality control screening, a total number of 203,224 polymorphic SNP markers were available for subsequent genome-wide association studies. These SNPs were distributed over all the 21 wheat chromosomes (Fig. 2). The above mentioned high-quality SNP markers were used to calculate linkage disequilibrium (LD) decay. The LD decay was measured as the physical distance at which LD dropped to half the maximum value. The LD decay distance was approximately 2.07 Mb (Fig. S1).

**Fig. 3:**
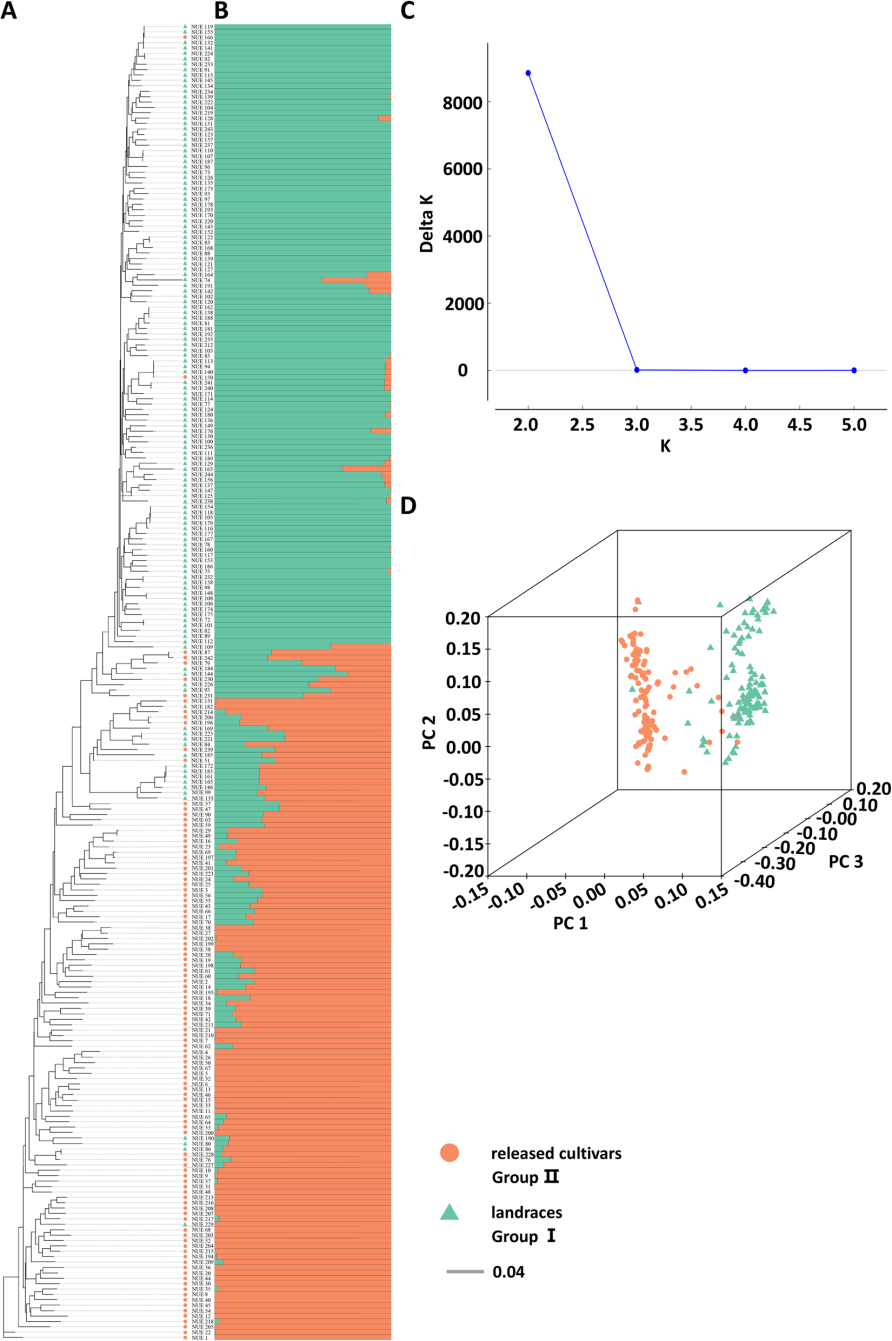
Population genomic analyses of 244 wheat accessions based on SNPs. **A** SNP-based phylogenetic tree. **B** SNP-based ADMIXTURE analysis at *K* = 2. **C** Determination of the number of subpopulations via the ad hoc statistic Δ*K*. **D** Principal component analysis

Population genetic structure analysis was based on all the 203,224 high-quality SNP markers for 244 wheat accessions. The results showed that a strong peak value of delta *K* (Δ*K*) appeared at *K* = 2 when the number of subpopulations changed from two to five, suggesting the optimal number of sub-populations as *K* = 2 (Fig. 3C, B). When *K* = 2, ancient landraces and released cultivars were separated two subpopulations, 1 (Group I) and 2 (Group II). In group I, 135 were landraces. In group II, 109 were released cultivars (Table S1). These observations were further supported by principal component analysis (PCA) (Fig. 3D) and phylogenetic tree construction (Fig. 3A). The above results indicated that the historic periods of release was the main factor for population structure delineation of wheat germplasms in Henan Province in this study.

### Identification of QTNs and QTN-by-environment interactions for three NUE-related traits using 3VmrMLM

**Fig. 4:**
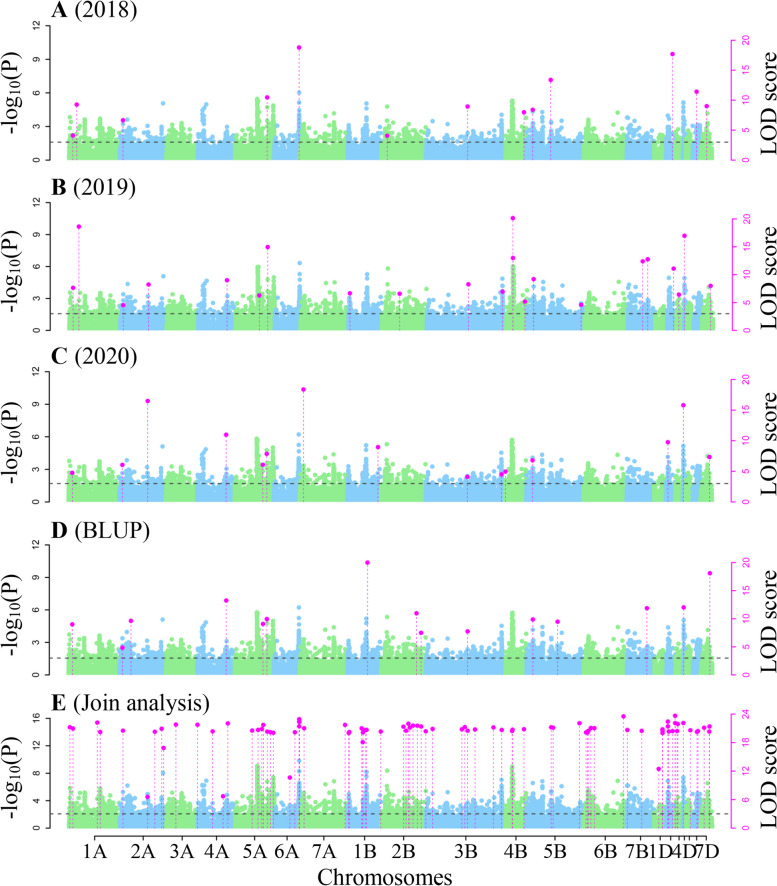
The Manhattan plot for grain yield per unit area ratio (GYAR) using software IIIVmrMLM. **A**–**C** QTNs detected for GYAR in 2018, 2019 and 2020 using single environment module in software IIIVmrMLM. **D** QTNs detected for GYAR BLUP values using single environment module in software IIIVmrMLM. **E** QTNs detected for GYAR across all the three environments using multi-environment joint analysis module in software IIIVmrMLM

**Fig. 5:**
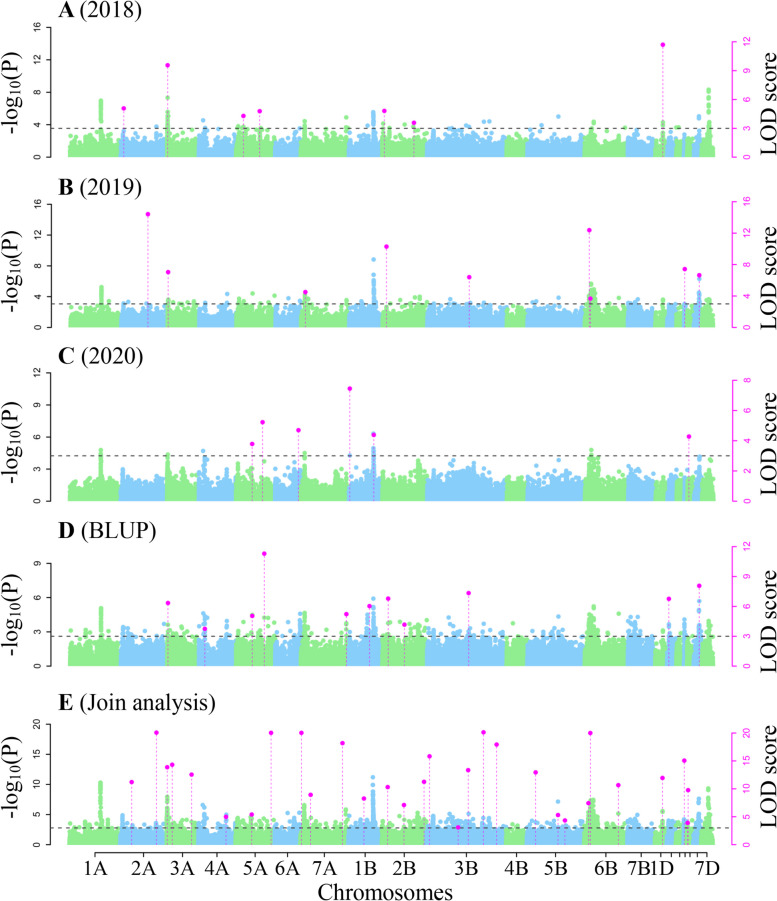
The Manhattan plot for effective panicle number ratio (EPNR) using software IIIVmrMLM. **A–C** QTNs detected for EPNR in 2018, 2019 and 2020 using single environment module in software IIIVmrMLM. **D** QTNs detected for EPNR BLUP values using single environment module in software IIIVmrMLM. **E** QTNs detected for EPNR across all the three environments using multi-environment joint analysis module in software IIIVmrMLM

**Fig. 6:**
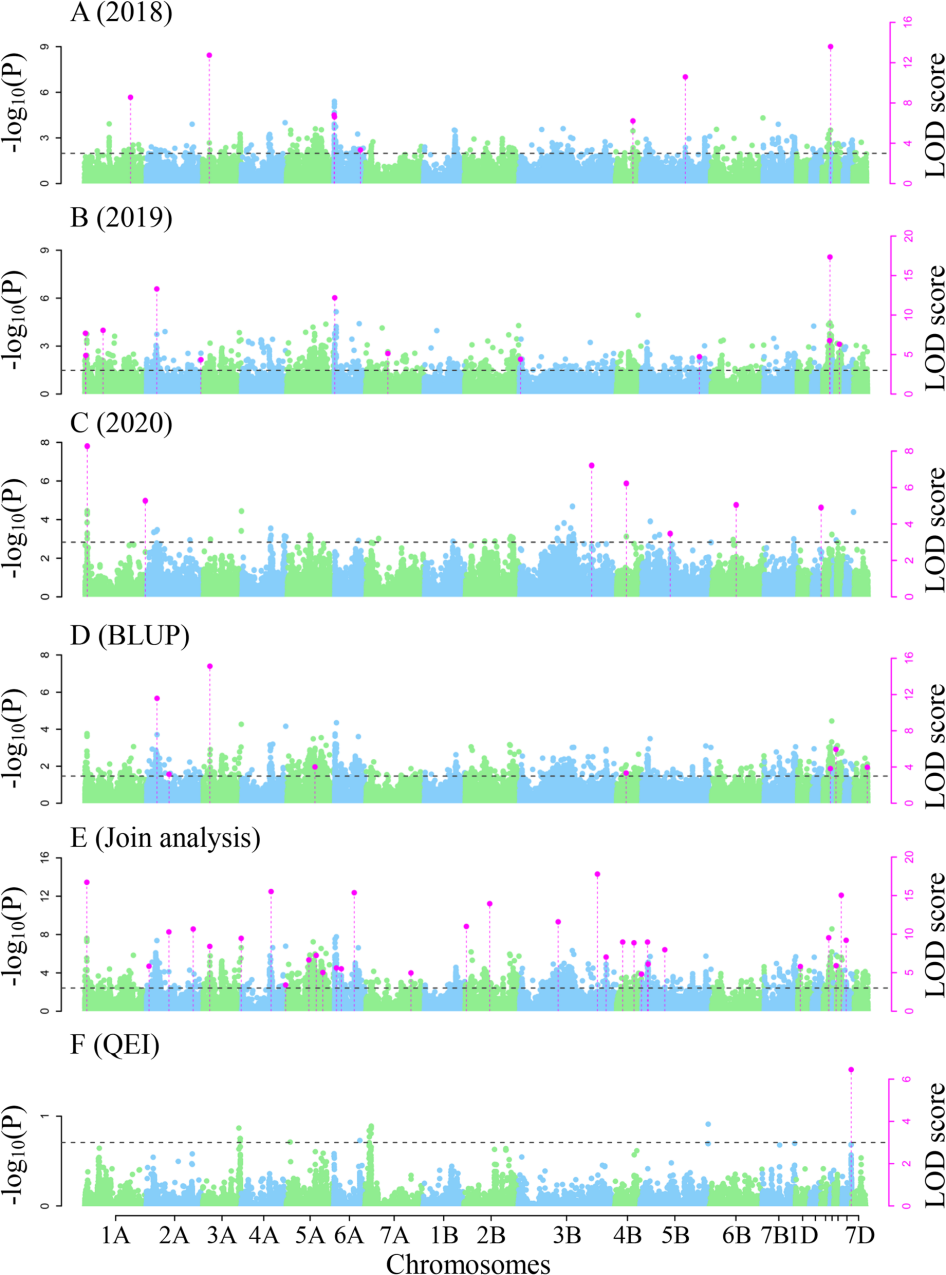
The Manhattan plot for plant height ratio (PHR) using software IIIVmrMLM. **A–C** QTNs detected for PHR in 2018, 2019 and 2020 using single environment module in software IIIVmrMLM. **D** QTNs detected for PHR BLUP values using single environment module in software IIIVmrMLM. **E** QTNs detected for PHR across all the three environments using multi-environment joint analysis module in software IIIVmrMLM. **F** QTN-by-environment interactions (QEIs) detected for PHR using multi-environment joint analysis module in software IIIVmrMLM

3VmrMLM was used to perform genome-wide association studies for all the three NUE-related traits. As a result, 133 QTNs were identified by single environment analysis (Table S3; Figs. 4A-D, 5A-D, and 6A-D), while 146 QTNs and one QEI were identified by multi-environment joint analysis (Tables S4-5; Figs. 4E, 5E, 6E-F). These loci were found to be significantly associated with NUE-related traits and distributed across all the 21 chromosomes. Among these loci, 33 were stably identified across multiple approaches, multiple environments, and multiple traits, these stable loci were distributed on all the chromosomes except 6A, 1B, 6B, and 5D, their proportions of total phenotypic variation.

explained by each QTN (*r*^2^) were from 0.13% to 16.59%, and their -log_10_ (*P*) values ranged from 3.20 to 211.56 (Table 2). In particular, 12 QTNs were identified in previous studies, while the remaining 21 were uncovered for the first time (Table 2). In this study, we focused on these new loci. The QTN AX-110463363 on chromosome 3AS with the largest *r*^2^, which we designated *Qphr3A.2*, was repeatedly identified in the PHR in 2018 (*r*^2^: 14.47%) and the BLUP value (*r*^2^: 16.59%), indicating the existence of a major locus around AX-110463363 for wheat NUE. Based on the stability of the SNPs with significant associations in each environment and the higher phenotype variation explanations, SNP AX-110463363 with largest effect was selected for subsequent candidate gene prediction. Importantly, genetic improvement simultaneously for nitrogen efficiency and plant height in wheat might be carried out by selecting for a single large-effect QTN.

**Table 2:** 33 stable QTNs detected in single- and multi-environment modules

QTN	Chr	Pos(Mb)	Marker	Trait	Environment^a^	Times	LOD	*r2*(%)	Sign	Know QTLs/QTNs/Genes	References
QTN 1	1A	30.18	AX-109023654	GYAR	1, 2, 3, 4, 5	5	4.10 ~ 116.84	0.77 ~ 2.96	SUG		
QTN 2	2A	561.28	AX-108728001	GYAR	2, 3	2	8.24 ~ 16.48	1.52 ~ 5.52	SIG		
QTN 3	2B	737.87	AX-111497324	GYAR	4, 5	2	7.49 ~ 158.51	1.19 ~ 2.40	SIG	qGNSR2B.3	Shi et al.,2022 [27]
QTN 4	2D	61.28	AX-109239751	GYAR	3, 5	2	9.76 ~ 161.15	0.96 ~ 2.87	SIG	Kukri_c16477_157	Zhao et al.,2022 [28]
QTN 5	2D	495.82	AX-111924626	GYAR	1, 2, 5	3	11.09 ~ 62.36	0.13 ~ 2.81	SIG		
QTN 6	3B	795.38	AX-109501776	GYAR	3, 5	2	4.51 ~ 86.11	0.28 ~ 1.32	SUG		
QTN 7	3D	515.93	AX-89699893	GYAR	2, 5	2	6.43 ~ 211.56	1.10 ~ 1.63	SUG	AX-109688668	Hu et al.,2020 [29]
QTN 8	4A	659.66	AX-110542298	GYAR	2, 3, 4	3	9.00 ~ 13.25	1.80 ~ 3.92	SIG		
QTN 9	4B	657.26	AX-109412797	GYAR	1, 2, 5	3	5.18 ~ 101.96	0.66 ~ 3.20	SIG	BobWhite_c8266_582	Zhao et al.,2022 [28]
QTN 10	4D	22.29	AX-89605902	GYAR	2, 3	2	15.80 ~ 16.98	2.40 ~ 3.63	SIG	*TaGS1.3*	Teng et al.,2022 [30]
QTN 11	5A	603.61	AX-109975954	GYAR	1, 2, 3, 4, 5	5	7.86 ~ 51.59	0.22 ~ 3.33	SIG		
QTN 12	5B	64.56	AX-110432662	GYAR	1, 2, 3, 4	4	6.79 ~ 9.88	2.16 ~ 3.18	SIG	MQTL5B.5	Saini et al.,2021 [31]
QTN 13	7B	498.97	AX-94561124	GYAR	2, 5	2	12.40 ~ 67.64	0.33 ~ 2.71	SIG		
QTN 14	7B	615.73	AX-108742467	GYAR	2, 4	2	11.88 ~ 12.77	1.27 ~ 1.54	SIG		
QTN 15	7D	572.02	AX-109531205	GYAR	2, 3, 4, 5	4	7.33 ~ 159.65	0.76 ~ 4.56	SIG		
QTN 16	2A	43.93	AX-109315961	GYAR, EPNR	1, 2, 3, 4, 5	5	4.52 ~ 71.48	0.38 ~ 4.44	SUG	Chr2A_SNP_03717	Lisker et al.,2022 [32]
QTN 17	2B	52.67	AX-109992665	GYAR, EPNR	1, 4, 5	3	4.06 ~ 10.31	1.27 ~ 4.22	SUG		
QTN 18	2B	450.75	AX-111609432	GYAR, EPNR	4, 5	3	4.16 ~ 154.47	0.94 ~ 2.50	SUG		
QTN 19	2B	642.83	AX-109282796	GYAR, EPNR	1, 5	2	3.58 ~ 177.95	1.48 ~ 3.23	SUG		
QTN 20	3B	376.86	AX-111689267	GYAR, EPNR	1, 2, 3, 4, 5	7	4.10 ~ 70.38	0.40 ~ 4.65	SIG		
QTN 21	5A	568.14	AX-108917610	GYAR, EPNR	3, 4, 5	4	6.07 ~ 192.52	1.61 ~ 5.24	SUG	qGNSR5A.2	Shi et al.,2022 [27]
QTN 22	7A	39.86	AX-110409398	GYAR, EPNR	2, 5	2	4.50 ~ 122.15	0.73 ~ 3.85	SUG		
QTN 23	1D	54.12	AX-109204935	EPNR	1, 5	2	11.70 ~ 11.95	0.87 ~ 3.43	SIG		
QTN 24	3A	1.93	AX-109519142	EPNR	1, 2, 5	3	7.04 ~ 13.87	2.39 ~ 8.34	SIG	BobWhite_c18256_105	Zhang et al.,2021 [33]
QTN 25	5A	452.44	AX-109874103	EPNR	3, 4, 5	3	3.79 ~ 5.40	0.81 ~ 3.98	SUG	qFsnR5A.2	Shi et al.,2022 [27]
QTN 26	6D	463.91	AX-110242843	EPNR	2, 4	2	6.65 ~ 8.07	4.95 ~ 5.77	SIG		
QTN 27	2A	100.63	AX-108992014	PHR	2, 4	2	11.58 ~ 13.30	9.17 ~ 11.05	SIG		
QTN 28	2A	330.96	AX-111237121	PHR	4, 5	2	3.20 ~ 10.28	2.07 ~ 3.11	SUG		
QTN 29	3A	67.37	AX-110463363	PHR	1, 4, 5	3	8.41 ~ 15.13	1.66 ~ 16.59	SIG		
QTN 30	3D	608.68	AX-110061772	PHR	2, 4	2	3.82 ~ 17.35	3.66 ~ 13.32	SIG	AX-109649974	Hu et al.,2020 [29]
QTN 31	4B	406.45	AX-110005628	PHR	3, 4	2	3.33 ~ 6.23	3.20 ~ 6.48	SUG		
QTN 32	4B	582.56	AX-111661088	PHR	1, 5	2	6.22 ~ 8.88	1.13 ~ 6.46	SUG		
QTN 33	4D	485.49	AX-111684216	PHR	4, 5	2	5.91 ~ 5.95	1.11 ~ 5.65	SUG	MQTL4D.2	Saini et al.,2021 [31]

*QTN* Quantitative trait nucleotide, *Chr* Chromosome, *Pos* Position, *PHR* Plant height ratio, *EPNR* Effective panicle number ratio, *GYAR* Grain yield per unit area ratio

^a^The five environments 2018, 2019, 2020, BLUP, and multiple-environment are marked as 1 to 5, respectively. Sign Significance; SIG significant (-log_10_ (*p*) ≥ 6.61); SUG suggestion (LOD ≥ 3)

### Prediction of NUE-related candidate genes

**Fig. 7:**
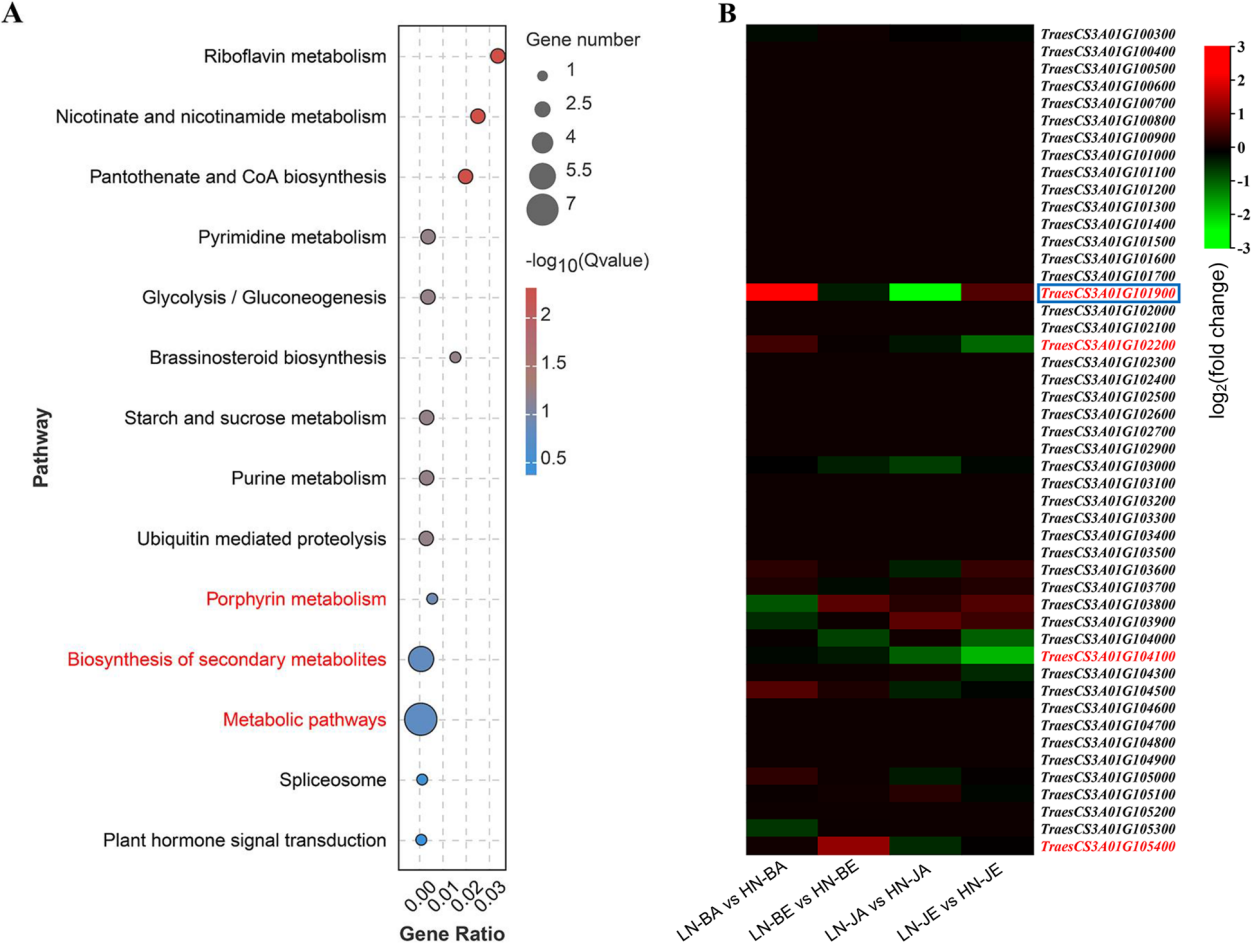
Candidate genes around qPHR3A.2. **A** KEGG enrichment analysis of 52 annotated genes. The pathways enriched for the target genes are in red font. The KEGG image used in this figure is licensed by KEGG copyright and has been changed accordingly. **B** Fold change of 52 candidate genes. The red, black, and green represent higher in low N than high N of a particular accession at a particular growth stage, close to high N, and lower than high N of a particular gene, respectively. Differentially expressed genes are in red font. Target genes are in blue boxes. LN low N treatment, HN high N treatment, B Bainong 207, J Jinzihong, A flowering stage, E 15 days past anthesis

Based on the LD decay distance of 2.07 Mb in Fig. S1, 2.07 Mb on each side of the significant locus *qPHR3A.2* for NUE was used to mine NUE-related candidate genes from the Chinese spring version 1.0 genome (http://202.194.139.32/). As a result, there were 52 annotated genes in the region (Table S6). Further KEGG enrichment analysis showed that 11 of them could be functionally enriched mainly in the metabolic, genetic information processing and environmental information processing pathways in common wheat, especially, there were seven metabolism-related candidate genes (Fig. 7A and Fig. S2) ([34–36], https://www.kegg.jp/kegg/kegg1.html).

To further determine candidate genes, transcriptome analysis was performed on all the 52 annotated genes using cultivars with different low nitrogen tolerance (Table S7). Results showed that four of them were differentially expressed after induction, i.e., the expression levels were significantly changed in at least one accession with different N treatments, indicating that the four genes were transcriptionally induced by the LN treatment (Fig. 7B; Table S6). Among the four DEGs, two were related in metabolic pathways in the analysis of KEGG and referred as candidates for NUE-related genes (Fig. S2; Table S6). In detail, *TraesCS3A01G102200* was differentially expressed only in Jinzihong at 15 DPA (LN-JE vs HN-JE). However, *TraesCS3A01G101900* was differentially expressed between the HN and LN treatments both in Bainong 207 and Jinzihong at anthesis (LN-BA vs HN-BA and LN-JA vs HN-JA). Although *TraesCS3A01G101900* was no differentially expressed in these two varieties at 15 DPA (LN-BE vs HN-BE and LN-JE vs HN-JE), *TraesCS3A01G101900* was down-regulated and up-regulated expressed at 15 DPA in Bainong 207 and Jinzihong (LN-BE vs HN-BE and LN-JE vs HN-JE), respectively. Among the two candidates, *TraesCS3A01G101900* was annotated in KEGG as a chlorophyllase and encoded hydrolase superfamily structural domain protein in NCBI conserved structural domain analysis, which was found in recently previous studies to be widely involved in abiotic stress processes [37]. In conclusion, *TraesCS3A01G101900* was regarded as preferred candidate gene of *qPHR3A.2*, named *TaCLH2*.

## Discussion

### Reliable results and significant progress

GWAS for wheat NUE in 244 landraces and released varieties (lines) in Henan Province, China provides new insight into the genetic basis of the important quantitative trait and varietal breeding. This study made reference to the results of Xing et al., 2022, and meet the increasing demand for wheat production, PHR, EPNR, and GYAR at wheat mature stage were used for GWAS. The results of GWAS showed that the reported NUE-related genes/QTLs (qGNSR2B.3, *TaGS1.3*, MQTL5B.5, MQTL4D.2) could be detected repeatedly in various environment. Among them, *TaGS1.3*, which was directly associated with NUE, were repeatedly detected in different environments in this study [12]. In summary, this analysis shows that these three traits can be taken as the indicator of NUE.

The 3VmrMLM model achieves very good performance, e.g., high power and accuracy, low false positive rate, and correct detection of all types of QTNs [26, 35]. RNA-seq has become an effective technique to examine genome-wide gene expression level [38]. Nevertheless, a very large set of DEGs is typically obtained, which made it difficult to identify underlying interesting candidate genes. Moreover, combined 3VmrMLM with RNA-seq has been recently used to predict candidate genes in soybean [39], rice [40], maize [41], and others. In the present study, a new comprehensive GWAS method, 3VmrMLM, was used for detecting QTNs, and QEIs of NUE-related traits under various nitrogen treatments. As a result, 12 stable QTNs had been reported in Wheat (Table 2).

In our study, 279 QTNs and 1 QEI for NUE-related traits were identified in three environments (Tables S3-5; Figs. 4, 5 and 6). Among these, 33 were repeatedly identified, in which 21 were new. Furthermore, one new candidate genes were mined by differential expression and KEGG enrichment analysis in the 2.07 Mb region flanking a large QTN (*r*^2^ > 10%), *qPHR.3A.2*. (Table S6; Fig. 7, Fig. S2).

### Comparison of N-efficient loci with reported loci in mature wheat

To investigate the relationship between the 33 replicated NUE SNP markers detected at maturity and the reported wheat NUE loci, a literature search revealed that 12 of the SNP markers may be closely linked to previously reported NUE loci and 21 markers may represent new NUE loci. Comparison revealed that AX-109315961, which was associated with relative effective spike per unit area and relative seed yield per unit area on chromosome 2AS in this study, might be related to Chr2A_SNP_03717, which has been reported to be associated with seed yield at field maturity. This association occurs because the two loci, SNP marker AX-109315961 and the previously reported Chr2A_SNP_03717, are approximately positioned 479,000 bp from each other on the Chinese spring reference genome [32]. SNP AX-109519142 on chromosome 3AS is 574 kb away from the previously reported BobWhite_c18256_105, as a consequence, SNP AX-109519142 is identical to BobWhite_c18256_105 [33]. SNP marker AX-109874103 on chromosome 5AL is the same locus as *qFsnR5A.2*, because AX-109874103 is located between SNP markers AX-110964357 and AX-109942297, which are closely linked to *qFsnR5A.2* [27]. AX-108917610 on chromosome 5AL is the same locus as the *qGNSR5A.2* NUE locus, as this locus is located both between the SNPs AX-109948410 and AX-111152617 that are tightly linked to both sides of *qGNSR5A.2* [27].

AX-111497324 on chromosome 2BL is at the same locus as *qGNSR2B.3* because AX-111497324 is located between markers AX-109374416 and AX-109848914 that are linked to both sides of *qGNSR2B.3* [27]. The SNP AX-109412797 on chromosome 4BL is 83 kb away from BobWhite_c8266_582, and therefore AX-109412797 is considered to be the same locus as BobWhite_c8266_582 [28]. SNP AX-110432662 on chromosome 5BS is identical to *MQTL5B.5* because SNP AX-110432662 is located between SNP markers AX-110612519 and AX-109473183, which are closely linked to MQTL5B.5 [31]. The SNP marker AX-109239751 on chromosome 2DS is 695 kb away from Kukri_c16477_157, and given this, AX-109239751 and Kukri_c16477_157 are also considered to be the same locus [28]. SNP AX-89699893 on chromosome 3DL is the same locus as AX-109688668, as SNP AX-89699893 is 1.59 Mb away from AX-109688668 [29]. Similarly, SNP AX-110061772 on chromosome 3DL is 0.32 Mb away from the reported SNP AX-109649974, so we consider these 2 loci as the same locus [29]. SNP AX-89605902 on chromosome 4DS is tightly linked to the wheat glutamine synthetase gene *TaGS1.3*, as this locus is only 660 kb away from *TaGS1.3* (*TraesCS4D02G047400*) [30]. The SNP marker AX-111684216 on chromosome 4DL is located between SSR Xwmc399, which is closely linked to MQTL4D.2, and SNP AX-109334705, so AX-110242843 is considered to be the same locus as MQTL4D.2 [31].

### Future prospects

A review of the literature revealed that 12 of the 33 replicate detected QTNs were closely linked to reported QTLs, loci or genes significantly associated with NUE and NUE-related traits in wheat. A novel locus on chromosome 3A, SNP AX-110463363, had an explanation rate between 1.66%-16.59%, indicating that genes affecting NUE are likely to be present on chromosome 3A. Next, we performed gene annotation of the candidate interval qPHR3A.2 for the new locus of NUE, and a preferred candidate gene encoding chlorophyllase was detected on chromosome 3A (AX-110463363). The hydrolase, chlorophyllase, is responsible for the breakdown of chlorophyll a, thereby generating dephyllophyll a and phytol which is known to play important roles in plant fruit ripening, leaf senescence, and processes resulting from biotic and abiotic stresses.

It has been suggested that citrus CLH plays a central role in chlorophyll degradation during decolorization of citrus fruits [42]. Hu et al. (2015) suggested that CLH is involved in secondary defense of Arabidopsis against herbivorous insects [43]. In a study on the effects of nitrogen fertilization on leaf senescence and carbon and nitrogen metabolism in late maize reproduction, Du et al. (2020) found that under suitable nitrogen fertilization conditions, the CLH activity of cob leaves was relatively low and chlorophyll content decreased slowly, which was conducive to the prevention of premature leaf senescence in cob leaves [44]. Zhang et al. (2020) found significant up-regulation of expression of genes encoding chlorophyllase in transcriptome analysis of N reduction on leaves of potato varieties with different N efficiency [45]. A study by Tian et al. (2021) showed that in Arabidopsis *CLH1* plays an important role in young leaf photoprotection by activating chlorophyll degradation activity through N-terminal shear post-translational modification, degrading chlorophyll and assisting chloroplast protease-mediated degradation of PSII core protein [46].

## Conclusion

Thirty-three significant QTNs were stably identified across different traits, environments, and approaches using compressed variance component mixed model method, 12 previously reported loci confirmed the reliability of the results in this study, more importantly, 21 novel loci were identified. Around the locus qPHR3A2, *TaCLH2* (*TracesCS3A01G101900*) was found in KEGG and gene expression analysis to be associated with NUE, although its biological function and molecular mechanism required further research in the future.

## Methods

### Plant material

The association panel was comprised of 135 landraces in 1940s and 109 improved cultivars from 1940 to 2010s (main cultivars, backbone parents, and pioneer varieties), and their seeds were provided by Henan Academy of Crops Molecular Breeding. The formal identification of the plant material used in this study was performed by Professor Weigang Xu of Henan Academy of Agricultural Sciences, and the voucher specimen of this material has been deposited in Institute of Crop Molecular Breeding, Henan Academy of Agricultural Sciences, Henan, China.

### Field trait measurement and statistical analysis

All the accessions were planted in three growing seasons in the Henan Research and Development Center for Modern Agriculture (Yuanyang County, Henan Province; 35◦ 00′ N 113◦ 40′ E, 77 m a.s.l.). All the trials were arranged in a completely randomized block design with three replications. Each plot consisted of four rows with 2 m in length and 0.23 m in width between rows, and sowing was conducted manually at a seeding rate of 130 seeds/m^2^ on October 11, 2017, October 10, 2019, and October 10, 2020. Two N treatments were applied: low N treatment (LN) and high N treatment (HN). The basic physicochemical characteristics and fertilization level are detailed in Table 1.

As described in Peng et al. (2021), PH, EPN, and grain yield per unit area (GYA) were measured [47]. The method described in Shi et al. (2022) was used to calculate low N tolerance for PH, GYA, EPN, and the best linear unbiased estimation (BLUP) [41]. As described in Tang et al. (2019), the processed data post normalization was used as the adjusted phenotypic values for genome-wide association studies [9]. For the zero mean normalization, the equation was

$${x}_{\rm Z}=\frac{x-\mu }{\sigma },$$

where ${x}_{\rm Z}$ is the zero mean normalized phenotypic values, $x$ is the relative phenotype values, $\mu$ is the phenotype mean, $\sigma$ is the standard deviation.

Descriptive statistical analysis and one-way ANOVA were performed using IBM SPSS v22.0 (SPSS, Chicago, IL, USA).

### Genotyping and marker screening

Total genomic DNAs of 244 accessions were extracted from fresh seedling leaves using a modified CTAB method, following Du et al. (2021) [48]. Genotyping for all 244 wheat accessions was carried out using SNP 660 K wheat microarray developed by Zhongyujin Marker Biotechnology Co., Ltd., Beijing, China. To reduce errors, SNP markers with minor allele frequency (MAF) < 0.05, missing data > 0.1 and heterozygosity > 0.1 were removed.

### Population structure and linkage disequilibrium (LD) analysis

The population structure of 244 wheat accessions was determined using the STRUCTURE v2.3.4 software [49]. The number of subgroups (*K*) was set from 2 to 5. We set BURNIN = 10,000 and NUMREPS = 110,000, respectively, and ran 5 replicates for each value of *K*. The optimal *K* value based on the Log-likelihood method was calculated with STRUCTURE HARVESTER version 0.6.94 (https://taylor0.biology.ucla.edu/structureHarvester/). Phylogenetic tree was constructed by the neighbor-joining method using PHYLIP version 3.698 (http://evolution.gs.washington.edu/phylip.html). Principal component analysis (PCA) was implemented via the PLINK-v1.07 software (http://zzz.bwh.harvard.edu/plink/download.shtml#download). The LD pattern between the pairs of SNPs was computed using PopLDdecay version 3.42 software (https://github.com/BGI-shenzhen/PopLDdecay/releases/tag/v3.42).

### Genome-wide association studies

Q matrix produced by Structure was included as covariate in the analysis to control for potential populations structure. To control polygenic background, marker inferred kinship matrix K was calculated by the 3VmrMLM software [26]. The single environment module of the 3VmrMLM method was used to identify QTNs for three NUE-related traits in each environment, while multi-environment module was used to detect QTNs and QTN-by-environment interactions [50]. As described in Li et al. (2022b), the critical *P*-value and LOD thresholds were set as 0.05/m and 3.0, respectively, for significant and suggestive QTNs, where m is the number of markers [50].

### Identification of candidate genes

Based on the LD attenuation distance, the related genes in a flanking region of the physical location of the most significant/suggested loci were annotated using wheat reference genome (CS RefSeq version 1.0) [51].

Bainong 207 (Low-N sensitive) and Jinzihong (low-nitrogen-tolerant) were used as the materials of RNA-seq analysis, and the field experiments were described as previous ones. At the anthesis stage, the main shoots of the plants was tagged. 10 flag leaves of each plot was randomly sampled at anthesis, 15 days past anthesis (DPA). Samples were quick frozen in liquid nitrogen after sampling and stored at − 80 °C. Total RNA extraction, mRNA library preparation, and Illumina sequencing were entrusted to Guangzhou Genedenovo Biotechnology Co., Ltd. (Guangzhou, China).

Differential expression analysis and KEGG enrichment analysis were carried out on with the Omicsmart website (https://www.omicsmart.com) and KEGG databases [34–36]. Expected number of Fragments Per Kilobase of transcript sequence per Millions base pairs sequenced (FPKM) was used to evaluate genes expression level. Only expressed genes with mean FPKM ≥ 1 were considered. False discovery rate (FDR) ≤ 0.05 and |log_2_ (fold change)|≥ 1 were considered as screening criteria for differentially expressed genes (DEGs). The heatmap module in TBtools (v1.120) was used to cluster and draw the heat map [52].

All methods were carried out in accordance with relevant guidelines.

### Supplementary Information

**Additional file 1:** **Table S1.** Information of 244 wheat accessions. **Table S2.** Summary statistics of BLUP of agronomic traits. **Table S3.** QTNs for wheat NUE-related traits in a single environment using 3VmrMLM. **Table S4.** QTNs for wheat NUE-related traits in all the environments using the 3VmrMLM. **Table S5.** QEIs for wheat NUE-related traits in all the environments using 3VmrMLM. **Table S6.** 52 Annotated genes within the confidence interval of SNP AX-110463363. **Table S7.** The phenotypic data for Bainong 207 and Jinzihong under low and high N conditions.

**Additional file 2:** **Figure S1.** Plot of *r*^2^ against distance between a pair of single-nucleotide polymorphisms (SNPs) in 244 wheat accessions. **Figure S2.** Distribution of the differentially expressed genes (DEGs) and response to nitrogen metabolism genes (NR).

## Data Availability

RNA sequencing data reported in this study have been deposited in the Genome Sequence Archive (GSA) database in China National Center for Bioinformation (CNCB) under accession number CRA013522 (https://ngdc.cncb.ac.cn/gsa). The genotypic and phenotypic data that support the findings of this study are available from the corresponding author upon reasonable request.

## Abbreviations

NUE: Nitrogen use efficiency
QTNs: Quantitative trait nucleotides
QTLs: Quantitative trait loci
GYA: Grain yield per unit area
NupE: Nitrogen uptake efficiency
NutE: Nitrogen utilization efficiency
EPN: Effective panicle number per unit area
PH: Plant height
LD: Linkage disequilibrium
PCA: Principal component analysis
MAF: Minor allele frequency
BLUP: Best linear unbiased estimation
DPA: Days past anthesis
FPKM: Fragments Per Kilobase of transcript sequence per Millions base pairs
DEGs: Differentially expressed genes
FDR: False discovery rate

## References

[1] Mourad A, Belamkar V, Baenziger PS (2020). Molecular genetic analysis of spring wheat core collection using genetic diversity, population structure, and linkage disequilibrium. BMC Genomics.

[2] Hao Y, Hao M, Cui Y, Kong L, Wang H (2022). Genome-wide survey of the dehydrin genes in bread wheat (*Triticum aestivum L.*) And its relatives: identification, evolution and expression profiling under various abiotic stresses. BMC Genomics.

[3] Rabieyan E, Bihamta MR, Moghaddam ME, Mohammadi V, Alipour H (2022). Genome-wide association mapping and genomic prediction of agronomical traits and breeding values in Iranian wheat under rain-fed and well-watered conditions. BMC Genomics.

[4] Mourad AMI, Alomari DZ, Alqudah AM, Sallam A, Salem KFM. Recent advances in wheat (Triticum spp.) Breeding. In: Al-Khayri JM, Jain SM, Johnson DV, editors. Advances in Plant Breeding Strategies: Cereals: Springer; 2019. 559–93.

[5] Zhao C, Ma G, Zhou L, Zhang S, Su L, Sun X, Borras-Hidalgo O, Li K, Yue Q, Zhao L (2021). Effects of nitrogen levels on gene expression and amino acid metabolism in Welsh onion. BMC Genomics.

[6] Chen C, Chu Y, Huang Q, Zhang W, Ding C, Zhang J, Li B, Zhang T, Li Z, Su X (2021). Morphological, physiological, and transcriptional responses to low nitrogen stress in populus deltoides marsh. Clones with contrasting nitrogen use efficiency. BMC Genomics..

[7] Pace J, Gardner C, Romay C, Ganapathysubramanian B, Lubberstedt T (2015). Genome-wide association analysis of seedling root development in maize (*Zea mays L.*). BMC Genomics.

[8] He Y, Xi X, Zha Q, Lu Y, Jiang A (2020). Ectopic expression of a grape nitrate transporter vvnpf6.5 improves nitrate content and nitrogen use efficiency in Arabidopsis. BMC Plant Biol.

[9] Tang W, Ye J, Yao X, Zhao P, Xuan W, Tian Y (2019). Genome-wide associated study identifies nac42-activated nitrate transporter conferring high nitrogen use efficiency in rice. Nat Commun.

[10] Monostori I, Szira F, Tondelli A, Árendás T, Gierczik K, Cattivelli L, Galiba G, Vágújfalvi A. Genome-wide association study and genetic diversity analysis on nitrogen use efficiency in a central European winter wheat (*Triticum aestivum L.*) Collection. PLoS One. 2017;12(12):e0189265. 10.1371/journal.pone.0189265 PMC5746223 29283996

[11] Jiang L, Sun L, Ye M, Wang J, Wang Y, Bogard M, Lacaze X, Fournier A, Beauchêne K, Gouache D, Wu R (2019). Functional mapping of n deficiency-induced response in wheat yield-component traits by implementing high-throughput phenotyping. Plant J.

[12] Xing P, Zhang X, Li D, Wang H, Bao Y, Li X (2022). Genome-wide association study identified novel genetic loci controlling internode lengths and plant height in common wheat under different nitrogen treatments. Euphytica.

[13] Cui Z, Luo J, Qi C, Ruan Y, Li J, Zhang A, Yang X, He Y (2016). Genome-wide association study (GWAS) reveals the genetic architecture of four husk traits in maize. BMC Genomics.

[14] Le TD, Gathignol F, Vu HT, Nguyen KL, Tran LH, Vu H, et al. Genome-wide association mapping of salinity tolerance at the seedling stage in a panel of Vietnamese landraces reveals new valuable QTLs for salinity stress tolerance breeding in rice. Plants (Basel). 2021;10(6):1088. 10.3390/plants10061088 PMC8228224 34071570

[15] Chen SY, Su MH, Kremling KA, Lepak NK, Romay MC, Sun Q, Bradbury PJ, Buckler ES, Ku HM (2020). Identification of miRNA-eQTLs in maize mature leaf by GWAS. BMC Genomics.

[16] Moussa AA, Mandozai A, Jin Y, Qu J, Zhang Q, Zhao H (2021). Genome-wide association screening and verification of potential genes associated with root architectural traits in maize (*Zea mays L.*) At multiple seedling stages. BMC Genomics.

[17] Fei X, Wang Y, Zheng Y, Shen X EL, Ding J, Lai J, Song W, Zhao H. Identification of two new QTLs of maize (*Zea mays L*.) Underlying kernel row number using the hnau-nam1 population. BMC Genomics. 2022;23(1):593. 10.1186/s12864-022-08793-1 PMC9380338 35971070

[18] Delfan S, Bihamta MR, Dadrezaei ST, Abbasi A, Alipour H (2023). Exploring genomic regions involved in bread wheat resistance to leaf rust at seedling/adult stages by using GWAS analysis. BMC Genomics.

[19] Eltaher S, Baenziger PS, Belamkar V, Emara HA, Nower AA, Salem K, Alqudah AM, Sallam A (2021). GWAS revealed effect of genotype x environment interactions for grain yield of Nebraska winter wheat. BMC Genomics.

[20] Moll RH, Kamprath EJ, Jackson WA (1982). Analysis and interpretation of factors which contribute to efficiency of nitrogen utilization. Agron J.

[21] Jin Y, Liu J, Liu C, Jia D, Liu P, Wang Y (2021). Genome-wide association study of nitrogen use efficiency related traits in common wheat (*Triticum aestivum L*.). Acta Agronomica Sinica.

[22] Guttieri MJ, Frels K, Regassa T, Waters BM, Baenziger PS (2017). Variation for nitrogen use efficiency traits in current and historical great plains hard winter wheat. Euphytica.

[23] Zhang J, Song Q, Cregan PB, Nelson RL, Wang X, Wu J, Jiang GL (2015). Genome-wide association study for flowering time, maturity dates and plant height in early maturing soybean (*Glycine max*) germplasm. BMC Genomics.

[24] Liu Y, Wang H, Jiang Z, Wang W, Xu R, Wang Q (2021). Genomic basis of geographical adaptation to soil nitrogen in rice. Nature.

[25] Yu J, Xuan W, Tian Y, Fan L, Sun J, Tang W (2021). Enhanced OsNLP4-OsNIR cascade confers nitrogen use efficiency by promoting tiller number in rice. Plant Biotechnol J.

[26] Li M, Zhang Y, Zhang Z, Xiang Y, Liu M, Zhou Y, Zuo J, Zhang H, Chen Y, Zhang Y (2022). A compressed variance component mixed model for detecting QTNs and QTN-by-environment and QTN-by-QTN interactions in genome-wide association studies. Mol Plant.

[27] Shi H, Chen M, Gao L, Wang Y, Bai Y, Yan H (2022). Genome-wide association study of agronomic traits related to nitrogen use efficiency in wheat. Theor Appl Genet.

[28] Zhao R. Nitrogen efficiency evaluation screening of wheat germplasm resources and genome-wide association analysis of related traits. Xianyang Shaanxi Province: Dissertation, Northwest A&F University; 2022.

[29] Hu C. Genome-wide association analysis of low nitrogen stress tolerance related traits in wheat seedling stage. Jinzhong Shanxi Province: Dissertation, Shanxi Agricultural University; 2020.

[30] Teng W, He X, Tong Y (2022). Genetic control of efficient nitrogen use for high yield and grain protein concentration in wheat: a review. Plants.

[31] Saini DK, Chopra Y, Pal N, Chahal A, Srivastava P, Gupta PK (2021). Meta-QTLs, ortho-MQTLs and candidate genes for nitrogen use efficiency and root system architecture in bread wheat (*Triticum aestivum L.*). Physiol Mol Biol Pla.

[32] Lisker A, Maurer A, Schmutzer T, Kazman E, Cöster H, Holzapfel J, Ebmeyer E, Alqudah AM, Sannemann W, Pillen K (2022). A haplotype-based GWAS identified trait-improving QTL alleles controlling agronomic traits under contrasting nitrogen fertilization treatments in the magic wheat population WM-800. Plants.

[33] Zhang P, Zhou X, Liang X, Guo Y, Zhao Y, Li S, Kong F (2021). Genome-wide association analysis for yield and nitrogen efficiency related traits of wheat at maturity stage. J Plant Nutr.

[34] Hiroyuki Ogata SGKS (1999). KEGG: Kyoto encyclopedia of genes and genomes. Nucleic Acids Res.

[35] Kanehisa M (2019). Toward understanding the origin and evolution of cellular organisms. Protein Sci.

[36] Kanehisa M, Furumichi M, Sato Y, Kawashima M, Ishiguro-Watanabe M (2023). Kegg for taxonomy-based analysis of pathways and genomes. Nucleic Acids Res.

[37] Li Q. Functional analysis of salicylic acid and its regulation-related genes (SABP2、SAMT) in plant stress tolerance. Tianjin: Dissertation, Tianjin University; 2019.

[38] Tai H, Lu X, Opitz N, Marcon C, Paschold A, Lithio A, Nettleton D, Hochholdinger F (2016). Transcriptomic and anatomical complexity of primary, seminal, and crown roots highlight root type-specific functional diversity in maize (*Zea mays L.*). J Exp Bot.

[39] Zuo JF, Chen Y, Ge C, Liu JY, Zhang YM (2022). Identification of QTN-by-environment interactions and their candidate genes for soybean seed oil-related traits using 3vmrmlm. Front Plant Sci.

[40] Zhang J, Wang S, Wu X, Han L, Wang Y, Wen Y. Identification of QTNs, QTN-by-environment interactions and genes for yield-related traits in rice using 3VmrMLM. Front Plant Sci. 2022;13:995609. 10.3389/fpls.2022.995609 PMC9618716 36325550

[41] Xiong X, Li J, Su P, Duan H, Sun L, Xu S (2023). Genetic dissection of maize (*Zea mays L.*) Chlorophyll content using multi-locus genome-wide association studies. BMC Genomics.

[42] Azoulay-Shemer T, Harpaz-Saad S, Cohen-Peer R, Mett A, Spicer V, Lovat N (2011). Dual n- and c-terminal processing of citrus chlorophyllase precursor within the plastid membranes leads to the mature enzyme. Plant Cell Physiol.

[43] Hu X, Makita S, Schelbert S, Sano S, Ochiai M, Tsuchiya T, Hasegawa SF, Hörtensteiner S, Tanaka A, Tanaka R (2015). Reexamination of chlorophyllase function implies its involvement in defense against chewing herbivores. Plant Physiol.

[44] Du L. Effects of density and nitrogen fertilizer on leaf senescence and carbon and nitrogen metabolism of summer maize at later growth stages in hilly region of Sichuan. Ya'an Sichuan Province: Dissertation, Sichuan Agricultural University; 2020.

[45] Zhang N, Zhang X, Song L, Su Q, Zhang S, Liu J (2020). Identification and validation of the superior alleles for wheat kernel traits detected by genome-wide association study under different nitrogen environments. Euphytica.

[46] Tian Y, Zhong R, Wei J, Luo H, Eyal Y, Jin H (2021). Arabidopsis chlorophyllase 1 protects young leaves from long-term photodamage by facilitating FtsH-mediated D1 degradation in photosystem II repair. Mol Plant.

[47] Peng C, Zhang Z, Li Y, Zhang Y, Dong H, Fang Y, Han L, Xu W, Hu L (2021). Genetic improvement analysis of nitrogen uptake, utilization, translocation, and distribution in Chinese wheat in Henan province. Field Crop Res.

[48] Du X, Xu W, Peng C, Li C, Zhang Y, Hu L (2021). Identification and validation of a novel locus, qpm-3bl, for adult plant resistance to powdery mildew in wheat using multi-locus GWAS. BMC Plant Biol.

[49] Evanno G, Regnaut S, Goudet J (2005). Detecting the number of clusters of individuals using the software structure: a simulation study. Mol Ecol.

[50] Li M, Zhang Y, Xiang Y, Liu M, Zhang Y (2022). IIIVmrMLM: the R and C ++ tools associated with 3VmrMLM, a comprehensive GWAS method for dissecting quantitative traits. Mol Plant.

[51] IWGSC, Appels R, Eversole K, Stein N, Feuillet C, Keller B, et al. Shifting the limits in wheat research and breeding using a fully annotated reference genome. Science. 2018;361(6403):eaar7191. 10.1126/science.aar7191 30115783

[52] Chen C, Chen H, Zhang Y, Thomas HR, Frank MH, He Y, Xia R (2020). TBtools: an integrative toolkit developed for interactive analyses of big biological data. Mol Plant.

