# Supplementary material for: Genome-wide association study of agronomic traits related to nitrogen use efficiency in Henan wheat
Source: BMC Genomics. 2024 Jan 2;25:7. doi: 10.1186/s12864-023-09922-0 (PMC10759698; doi:10.1186/s12864-023-09922-0)
Supplement: Supplementary file 2 — Additional file 2: Figure S1. Plot of r2 against distance between a pair of single-nucleotide polymorphisms (SNPs) in 244 wheat accessions. Figure S2. Distribution of the differentially expressed genes (DEGs) and response to nitrogen metabolism genes (NR). [file 12864_2023_9922_MOESM2_ESM.docx]

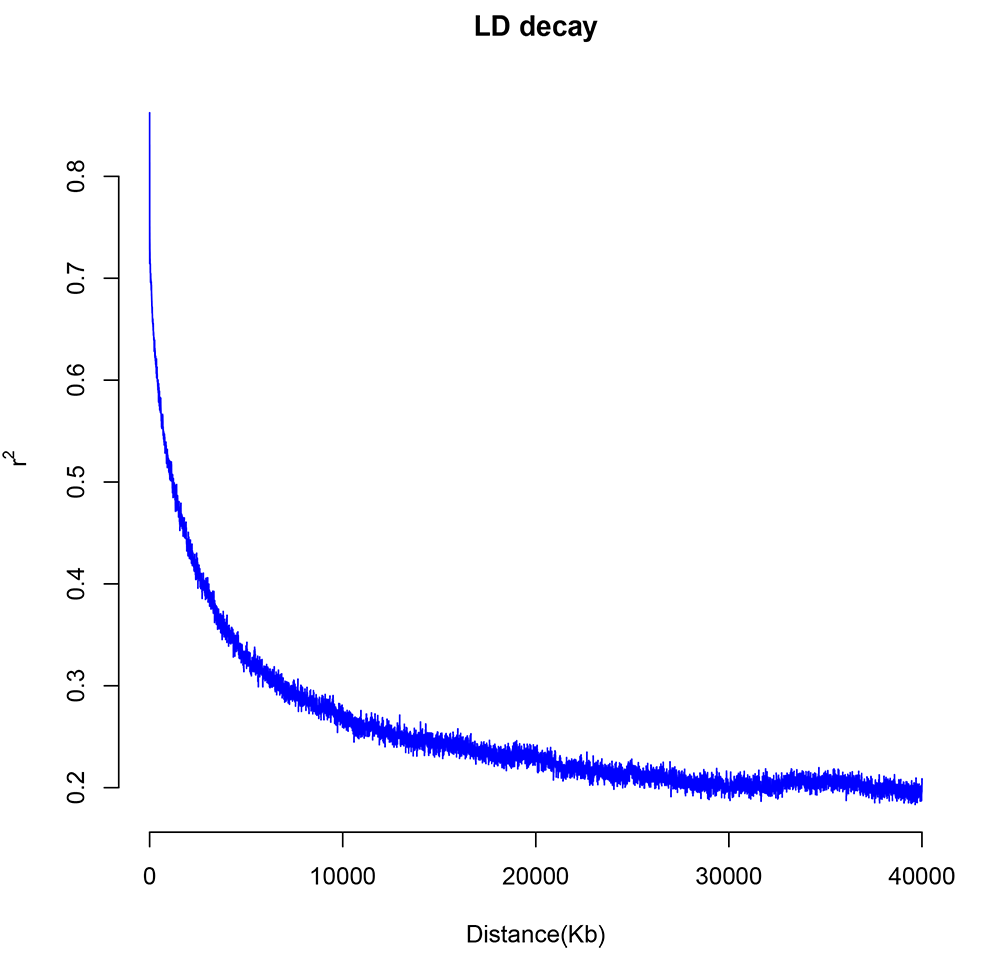


**Figure S1** Plot of *r*^2^ against distance between a pair of single-nucleotide polymorphisms (SNPs) in 244 wheat accessions.


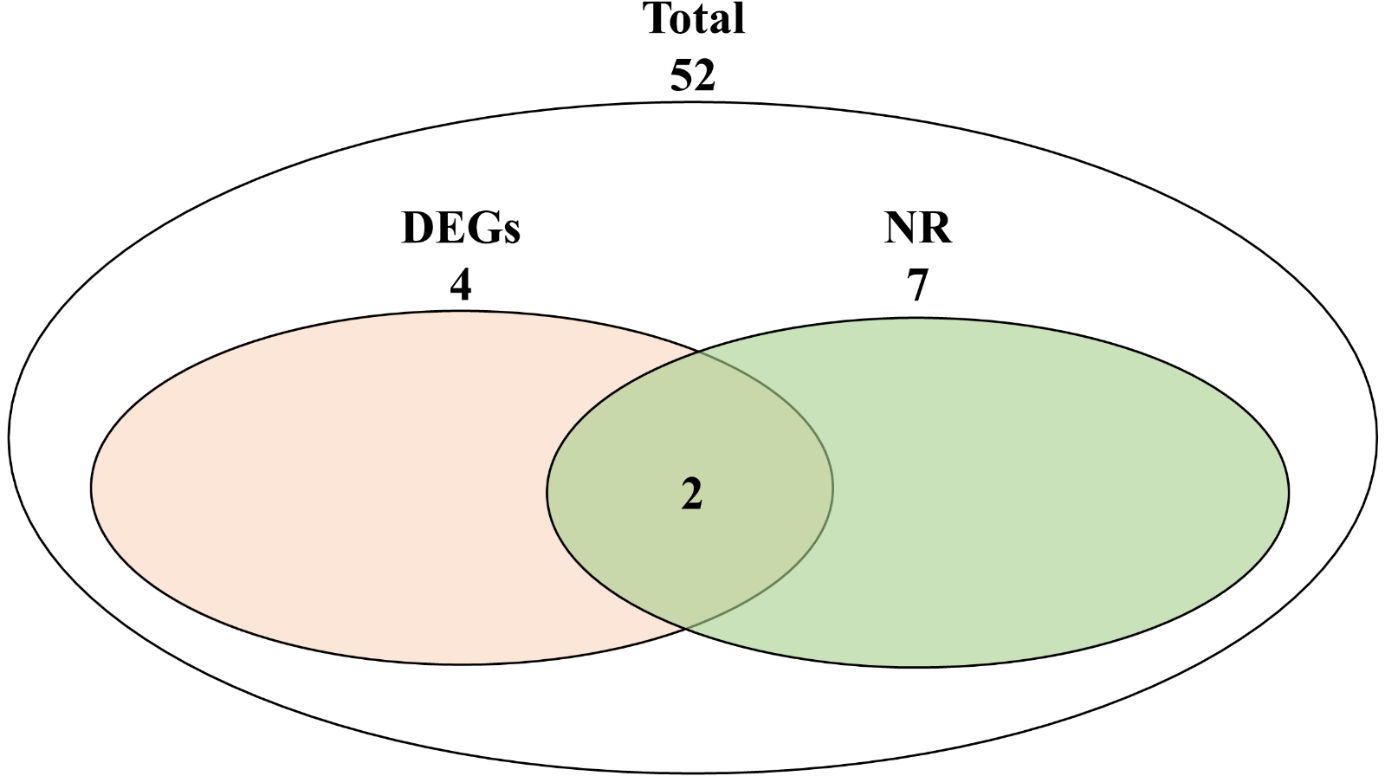


**Figure S2** Distribution of the differentially expressed genes (DEGs) and response to nitrogen metabolism genes (NR).
